# The diagnostic pathway to lymph node excision biopsy in an HIV- and tuberculosis-endemic region

**DOI:** 10.4102/sajhivmed.v26i1.1752

**Published:** 2025-10-31

**Authors:** Camagu Potelwa, Sheree Gray, Francois Malherbe, Christo Kloppers, David Richardson, Jenna Bailey, Karryn Brown, Estelle Verburgh

**Affiliations:** 1 Department of Medicine, Faculty of Health Sciences, University of Cape Town and Groote Schuur Hospital, Cape Town, South Africa; 2 Division of Clinical Haematology, Department of Medicine, Faculty of Health Sciences, University of Cape Town and Groote Schuur Hospital, Cape Town, South Africa; 3 Department of Surgery, Faculty of Health Sciences, University of Cape Town and Groote Schuur Hospital, Cape Town, South Africa

**Keywords:** HIV, tuberculosis, lymphadenopathy, lymphoma, diagnostic delay, fine needle aspiration, GeneXpert, South Africa

## Abstract

**Background:**

In the HIV/tuberculosis endemic Western Cape public care setting, diagnostic consideration of patients with persistent lymphadenopathy focuses on extra-pulmonary tuberculosis (EPTB), delaying diagnosis of other infectious or malignant causes of lymphadenopathy.

**Objectives:**

To evaluate the diagnostic pathways of patients investigated for lymphadenopathy at Groote Schuur Hospital.

**Method:**

A retrospective review of patients undergoing lymph node excision biopsy was conducted to correlate test selection and results prior to biopsy with final patient diagnosis and assess the impact of pre-biopsy pathways on diagnostic delay.

**Results:**

Of 86 patients undergoing excision biopsy, 61 (71%) had no previous diagnosis to explain the lymphadenopathy. Extra-pulmonary tuberculosis was the commonest diagnosis (24.6%, 15/61), followed by lymphoma (21.3%, 13/61), and metastatic cancer (14.8%, 9/61). Median time from presentation with lymphadenopathy to first excision biopsy was 55 days (interquartile range [IQR] 22–106). Fine needle aspiration (FNA) cytology of lymphadenopathy was performed in 30 out of 61 (49%) of the patients and repeated in a third of these, while smear for acid-fast bacilli and culture for *Mycobacterium tuberculosis* were infrequently performed, and the GeneXpert MTB/RIF assay on FNA was never performed. FNA cytology was non-diagnostic in all seven patients with lymphoma in whom it was performed.

**Conclusion:**

In patients with lymphadenopathy, we demonstrate how poorly structured diagnostic pathways contribute to unnecessary healthcare utilisation and diagnostic delay in treatable conditions. Providing early access to biopsy after appropriate workup ensures accurate diagnosis of unexplained lymphadenopathy.

**What this study adds:** This study highlights how suboptimal diagnostic pathways delay tuberculosis and lymphoma diagnosis in HIV-endemic settings. It underscores the limited value of FNA cytology and underuse of GeneXpert on FNA.

## Introduction

South Africa is the epicentre of the dual tuberculosis and HIV epidemics. Patients presenting with persistent lymphadenopathy and constitutional symptoms in the public healthcare system are deemed, in the first place, to suffer from extra-pulmonary tuberculosis (EPTB), regardless of age or HIV status.^1^ Less diagnostic consideration is given for malignant causes of lymphadenopathy, such as haematological malignancies, predominantly lymphoma, and solid cancer metastases to the lymph nodes.^2,3^

In people with HIV (PWH), EPTB accounts for up to 50% of tuberculosis cases, with tuberculosis adenitis the most common site of infection.^1,3,4^ The diagnosis of EPTB requires specific and sensitive tests to account for the pauci-bacillary nature of the disease. The introduction of the GeneXpert MTB/RIF assay (Xpert) enhanced diagnostic accuracy for active tuberculosis in lymph nodes and heralded an era of rapidly evolving role of molecular diagnostics. Subsequently, Xpert Ultra, and other proven tuberculosis nucleic acid amplification tests (TB-NAATs), have further improved test sensitivity, while point-of care testing further reduced time to diagnosis.^5,6,7^ Since Xpert’s inclusion in the 2013 WHO guidelines, TB-NAATs have been recommended for first-line assessment of EPTB.^8^ Despite this strong recommendation, TB-NAATs have not been widely implemented in the investigation of lymphadenopathy in our region.^9,10^ In common practice, lymphadenopathy is investigated using fine needle aspirate (FNA) sent for either cytology, tuberculosis microscopy, or both.^11^ The unacceptably low diagnostic accuracy of these tests leads to the failed detection of not only tuberculosis but also of lymphoma.^12,13^ With both conditions inaccurately ‘ruled out’, a presumptive diagnosis of EPTB often follows, with empiric tuberculosis therapy started without definitive proof of mycobacterial infection, and without clinical follow-up to ensure response.^1,3^

It is imperative that the clinician should not only consider EPTB as a differential diagnosis in patients with lymphadenopathy, but should also consider other diagnoses, notably lymphoma and solid cancer metastases. Considering these serious conditions as top differential diagnoses ensures that the clinician appropriately chooses the best tests to arrive at the correct diagnosis. Fine needle aspirate for cytology (FNAC) is a good first-line test for cancer, but has unacceptably low sensitivity for lymhoma and EPTB, which are more common, and more treatable.^3,12^ Antel et al. showed that the use of FNAC (especially repeated use) significantly delayed the diagnosis of lymphoma by increasing the interval from presentation to diagnosis, termed *the healthcare practitioner interval*.^1^

We hypothesised that the choice of tests performed in the diagnostic pathway of lymphadenopathy has an impact on diagnostic delay and increases the healthcare practitioner interval in patients referred for lymph node excision biopsies. We further wanted to analyse the tests related to the investigation of lymphadenopathy before excision biopsy, including tuberculosis investigations and tests related to aspiration of the lymph node. To investigate this, we conducted a retrospective review of all patients admitted to the surgical biopsy clinic at Groote Schuur Hospital (GSH) in 2016. These patients underwent a diagnostic excision biopsy of the lymph node, with the focus on obtaining a histological diagnosis.

## Research methods and design

### Study design and participant selection

GSH is a tertiary referral institution in Cape Town, South Africa. Patients aged 13 years and older, who are referred from peripheral hospitals/health centres or within GSH, are evaluated for excision of a palpable mass on the local anaesthetic day surgery list at GSH. Main presentations seen in this clinic are lymphadenopathy or soft tissue swelling (such as lipomas or sebaceous cysts). Patients are assessed clinically, and the need for an excisional biopsy is determined. The procedure is then performed under local anaesthesia, and histology results are communicated to patients during a follow-up visit or with a phone call. In this retrospective analysis, we included all patients who underwent a lymph node excision biopsy between 01 January 2016 and 31 December 2016. Patients who underwent soft tissue biopsies and those who did not qualify for a biopsy after clinical examination were excluded.

### Data collection

Data were sourced from the clinic booking register, hospital electronic and paper medical records and the National Health Laboratory Service (NHLS) laboratory information system called TrakCare (InterSystems, Cambridge, Massachusetts, United States).

#### Patient demographics

Age and sex were recorded for all participants.

#### Clinical characteristics

Medical history was recorded for all participants, including HIV and antiretroviral therapy (ART) status, and history of previously treated solid cancer, haematological malignancy or tuberculosis. The healthcare practitioner interval was determined by recording the interval between the date of first presentation with lymphadenopathy, and of the diagnostic excisional biopsy. The site of excisional biopsy was recorded (neck, axillary, inguinal, or other).

#### Laboratory investigations

All laboratory tests were conducted as part of routine clinical practice at the NHLS laboratory, which is accredited by the South African National Accreditation System and adheres to international quality standards (ISO 15189).

#### Investigations in the healthcare practitioner interval

All investigations to detect the presence of tuberculosis infection performed on sputum and FNA, such as Xpert (GeneXpert MTB/RIF; Hain Lifescience, Nehren, Germany), Ziehl-Neelsen stain for acid-fast bacilli (AFBs), and mycobacterial culture, with their respective results were recorded. All cytology results from FNAs were documented. For HIV-positive patients, the most recent CD4 counts (cells/mm^3^) and HIV viral load to categorise patients as virally suppressed (< 50 copies/mL) or unsuppressed were recorded.

#### Investigations on lymph node excision biopsies

Ziehl-Neelsen staining for AFBs, immunohistochemical and special stains were requested as indicated by the pathologist reviewing the case. Xpert and mycobacterial culture were performed if requested by the surgeon. Final histological diagnoses were used to categorise diagnoses as lymphoma (haematological malignancy), cancer, tuberculosis, other, or non-diagnostic. Lymphoma and cancer diagnoses were made according to the relevant WHO classification with multicentric Castleman’s disease included under lymphoma (new diagnostic category of ‘lymphoproliferations’).^14^ Patients with both definite tuberculosis (culture positive for *Mycobacterium tuberculosis*, and/or AFBs seen on FNA/tissue) and probable tuberculosis (no other diagnosis accounting for lymphadenopathy with macroscopic caseation, and/or granulomas seen, on FNA/tissue) were categorised as ‘tuberculosis’. All other benign lymph node pathologies were categorised as ‘other’. Cases were categorised as ‘non-diagnostic’ if the sample was insufficient for diagnosis or not representative of a lymph node.

#### Biopsy procedure

All lymph node excisions were performed according to standard surgical practice under local anaesthetic.

### Data analysis

Data analysis was performed using STATA Version 18 (StataCorp LP, College Station, Texas, United States; https://www.stata.com). Patients were analysed in two groups: ‘new diagnosis’ – those with no previous diagnosis to explain the lymphadenopathy; ‘previous diagnosis’ – those with a previous diagnosis of a haematological malignancy, cancer, or tuberculosis that required an excision biopsy for further characterisation, response assessments, or for suspected relapse. In the ‘new diagnosis’ group, patients were divided into five diagnostic outcome categories: lymphoma (haematological malignancy), cancer, tuberculosis, other, and non-diagnostic. Categorical variables were described by frequencies and percentages and compared across groups using Pearson’s Chi-squared or Fisher’s exact tests, as appropriate. Numerical variables were described by medians, and interquartile ranges (IQRs) as data were non-parametric. Numerical variables were compared across groups using Kruskal-Wallis tests.

### Ethical considerations

Ethical approval was obtained from University of Cape Town Human Research Ethics Committee (HREC: 829/2020) and GSH. A waiver of informed consent was granted as this was a retrospective study using anonymised data.

## Results

**FIGURE 1:**
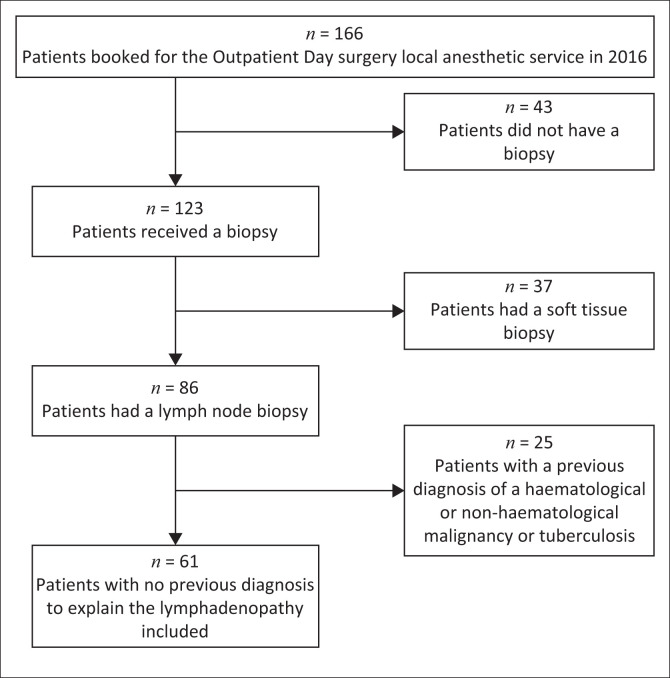
Flowchart showing participants included and excluded in this study.

In 2016, 166 patients were evaluated in the surgery clinic for ‘lumps and bumps’ (Figure 1). Of these patients, 86 had histological confirmation of a lymph node excision. The majority of these patients (71%, *n* = 61/86) had no previous diagnosis to explain the lymphadenopathy and were included in the study (Table 1 and Table 2). The remaining 25 patients, classified as ‘Previous diagnosis’, were undergoing repeat assessment for a previously confirmed haematological or non-haematological malignancy or tuberculosis and have been excluded from further analysis.

**TABLE 1:** The demographic and clinical characteristics of patients who had an excision biopsy for lymphadenopathy of an unknown cause (*N* = 61).

Variable	Diagnostic category						
	Total	Lymphoma (*n* = 13)†	Cancer (*n* = 9)	Tuberculosis (*n* = 15)	Other (*n* = 20)	Non-diagnostic (*n* = 4)	*P*
**Age at biopsy**							0.016*
Median	41	35	58	31	43.5	39	-
IQR	29–51	29–41	43–64	23–45	34.5–53	21–60.5	-
**Sex**							0.216
Male	25/61	7/13	6/9	6/15	5/20	1/4	-
Female	36/61	6/13	3/9	9/15	15/20	3/4	-
**HIV status**							0.136
HIV-positive	25/61	8/13	3/9	6/15	6/20	2/4	-
HIV-negative	30/61	4/13	5/9	8/15	13/20	0/4	-
Unknown	6/61	1/13	1/9	1/15	1/20	2/4	-
**CD4 count, cells/mm^3^ (*n* = 22)**							0.121
Median	181	278	264	83	400	129	
IQR	85–400	104–726	105–456	59–85	337–527	75–183	
**ART status of PWH**							0.104
On ART	17/25	7/8	1/3	4/6	4/6	1/2	-
Not on ART	5/25	1/8	2/3	2/6	0/6	0/2	-
Unknown	3/25	0/8	0/3	0/6	2/6	1/2	-
Suppressed HIV viral load for PWH on ART	11/24	7/8	0/3	0/6	3/6	1/2	0.002*
**Days in healthcare practitioner interval**							0.066
Median	55	32	74	74	79	18.5	-
IQR	22–106	14–44	49–107	35–92	27–156	11–171	-
**Days from sputum to biopsy (*n* = 31)**							0.143
Median	49	10.5	49	46	70	130	-
IQR	14–80	4–42	35–107	16–122	52–80	6–254	-
**Days from FNA to biopsy (*n* = 30)**							0.385
Median	30	10	30	74	91	17	-
IQR	6–86	5.5–44	6–34	24–86	0–235	2–94	-

Note: *P*-values calculated using Pearson’s Chi-squared/Fisher’s exact tests for categorical variables and Kruskal-Wallis tests for numerical variables.

IQR, interquartile range; ART, antiretroviral therapy; PWH, people with HIV; FNA, fine needle aspiration.

* , Statistically significant at *P* < 0.05.

† , Includes two patients with lymphoma and Kaposi sarcoma.

**TABLE 2:** Investigations performed prior to excision biopsy in patients with lymphadenopathy of an unknown cause (*N* = 61).

Variable	Total	Lymphoma (*n* = 13)†	Cancer (*n* = 9)	Tuberculosis (*n* = 15)‡	Other (*n* = 20)	Non-diagnostic (*n* = 4)§
**First sputum (*n* = 31)**						
GXP positive	2/26	0/4	0/7	2 /8	0/5	0/2
AFB positive	0/2	0/0	0/0	0/2	0/0	0/0
Tuberculosis culture positive	1/7	1/2	0/1	0/1	0/2	0/1
**Second sputum (*n* = 10)**						
GXP positive	1/9	0/2	1/2	0/3	0/1	0/1
Tuberculosis culture positive	1/1	0/0	0/0	1/1	0/0	0/0
**First FNA (*n* = 30)**						
AFB positive	0/12	0/3	0/3	0/5	0/1	0/0
Tuberculosis culture positive	0/4	0/1	0/1	0/2	0/0	0/0
Cytology†						
Atypical	6/29	1/7	1/5	2/9	0/5	2/3
Negative for malignancy	9/29	4/7	2/5	2/9	1/5	0/3
Cancer	3/29	1/7	2/5	0/9	0/5	0/3
Necrosis	1/29	0/7	0/5	1/9	0/5	0/3
Necrotising granulomatous inflammation	1/29	0/7	0/5	1/9	0/5	0/3
Granulomatous inflammation	1/29	0/7	0/5	0/9	1/5	0/3
Non-diagnostic	8/29	1/7	0/5	3/9	3/5	1/3
**Second FNA (*n* = 6)**						
AFB positive	0/3	0/1	0/1	0/1	0/0	0/0
Tuberculosis culture positive	1/1	0/0	0/0	1/1	0/0	0/0
Cytology						
Negative for malignancy	3/5	0/1	1/1	0/1	1/1	1/1
Necrotising suppurative lymphadenitis	1/5	1/1	0/1	0/1	0/1	0/1
Non-diagnostic	1/5	0/1	0/1	1/1	0/1	0/1

AFB, acid-fast bacilli; FNA, fine-needle aspiration; GXP, GeneXpert MTB/RIF assay.

† , Includes two patients with lymphoma and Kaposi sarcoma;

‡ , In the tuberculosis category, we include five patients who had probable tuberculosis;

§ , Non-diagnostic cytology includes not representative, suboptimal, and reactive.

### Demographics, clinical characteristics, and time to diagnostic biopsy

Of the new patients, the majority (59.0%, *n* = 36/61) were women, with a median age of 41 years (IQR 29–51) (Table 1). PWH constituted 41.0% (*n* = 25/61) of the group. Most PWH were on ART (68.0%, *n* = 17/25), and 45.8% (*n* = 11/24) were virally suppressed at the time of excision biopsy. The median time from presentation with lymphadenopathy to first excision biopsy was 55 days (IQR 22–106). The lymphoma group had the shortest median time from presentation, sputum analysis, and FNA to first excision biopsy compared to the other groups. The median time from sputum Xpert/*M. tuberculosis* culture to first excision was 49 days (IQR 14–80), and from FNA to biopsy, 30 days (IQR 6–86).

### Diagnostic categories, age, and HIV status

Diagnostic categories are represented in Table 1. The most common diagnostic outcome was tuberculosis (24.6%, *n* = 15/61), followed by lymphoma (21.3%, 13/61), and cancer (14.8%, *n* = 9/61). Additionally, one patient with lymphoma had an isolated positive tuberculosis culture on sputum, and one cancer patient had an isolated positive Xpert on sputum. The remainder of the biopsies were benign lesions, classified as ‘other’, or were non-diagnostic. The median age was highest in those with cancer, and was significantly lower and comparable in patients with lymphoma and tuberculosis. Patients with lymphoma had the highest rate of HIV positivity (61.5%, *n* = 8/13), and almost all were on ART and virally suppressed with a median CD4 count of 278 cells/mm^3^ (IQR 104–726). In contrast, patients with tuberculosis had the lowest median CD4 count of 83 cells/mm^3^ (IQR 59–85), and none were virally suppressed, although 66.7% (*n* = 4/6) were on ART.

### Diagnostic tests performed during the healthcare practitioner interval

The diagnostic tests performed during the diagnostic or healthcare practitioner interval leading up to excisional biopsy are presented in Table 2.

### Sputum prior to excision biopsy

Prior to excision biopsy, more than half of all patients, and two-thirds of those with a final diagnosis of tuberculosis, had at least one sputum analysis. Xpert was the most performed test and was positive in 8.6% of cases (*n* = 3/35). Culture for *M. tuberculosis* was seldom performed, and was positive in 2 out of 8 cases. Smear for AFBs was seldom performed and was non-diagnostic.

### Fine needle aspirate prior to excision biopsy

Half of the patients had at least one FNA, and 9.8% (*n* = 6/61) had a second FNA. Fine needle aspirate for cytology was almost always requested (96.7%, *n* = 29/30 of first FNAs, and 83.3%, *n* = 5/6 of second FNAs). Fine needle aspirate for cytology was not diagnostic in the lymphoma cases. In one lymphoma case, which also had Kaposi sarcoma (KS), the cytology showed an undifferentiated neoplasm but missed the lymphoma diagnosis. Staining for AFBs and culture for *M. tuberculosis* were performed less frequently, and Xpert was never used on the FNAC. Staining for AFBs did not detect any cases of tuberculosis, and there was only one positive tuberculosis culture on a second FNA. Cytological examination of two definite tuberculosis cases showed one case with necrosis and another with necrotising granulomatous inflammation. In a second case with necrotising granulomatous inflammation, tuberculosis could not be confirmed, and the case was diagnosed as *probable tuberculosis*.

## Results of excision biopsy (histology)

### Tuberculosis

Of the 15 tuberculosis cases, 10 were confirmed as definite tuberculosis, and five were classified as probable tuberculosis. Most study patients (95.1%, *n* = 58/61) did not undergo an Xpert test on the tissue, and none of the tuberculosis patients received an Xpert test. In seven cases, AFBs were found, six of whom had a final diagnosis of tuberculosis, and one a final diagnosis of tuberculosis and lymphoma. A tuberculosis culture was performed on only 39.4% (*n* = 24/61) of the biopsies. Of the five patients in the tuberculosis group who had a culture, only two had a positive result. Of the six patients with positive AFBs on histology, only one underwent a tuberculosis culture, which was also positive.

### Lymphoma

Among the 13 lymphoma cases, there were seven classical Hodgkin lymphoma, one Burkitt lymphoma, and five multicentric Castleman disease (MCD).

### Cancer

The nine metastatic cancer cases consisted of three adenocarcinomas, one seminoma, two KS, two neuroendocrine carcinomas, and one renal cell carcinoma. There were two patients classified under lymphoma that had additional diagnoses on histology, in one case MCD and *M. kansasii*, and the other had KS.

### Other

The ‘Other’ subgroup included 10 cases of dermatopathic lymphadenitis, five cases of reactive lymphoid hyperplasia, two cases of reactive lymph nodes, and one case each of follicular hyperplasia, HIV-related follicular hyperplasia, and fibrosis. Of the nine patients who had a second biopsy, only one was non-diagnostic.

## Discussion

We describe the laboratory investigations performed prior to excisional lymph node biopsy and how inappropriate test selection may contribute to diagnostic delay. Our retrospective analysis reveals that FNA was frequently performed but mostly utilised for cytology, a test with poor sensitivity for the main differential diagnosis of tuberculosis and lymphoma. The preferred rule-out test for tuberculosis on FNA, Xpert, was never performed. In this cohort, histological assessment of excisional biopsies showed that over 60% of patients had one of three notable diagnoses, namely: tuberculosis, lymphoma, and metastatic cancer. The first two conditions respond well to prompt treatment, but poorly to late treatment, underscoring the urgency of improving pathways to timely and accurate diagnosis of these lymphadenopathy disease differentials.

The gold standard histological diagnosis of lymphadenopathy is obtained through a surgical excision biopsy. In this cohort, it took a median of 55 days (IQR 22–106) from presentation with persistent lymphadenopathy to a diagnostic biopsy. According to the UK NICE guidelines, 28 days is an acceptable target from presentation to diagnosis when a patient presents with suspected haematological malignancy.^1,15^ These guidelines further specify that, ideally, a biopsy should be performed within 2–3 weeks. The recommended diagnostic interval was not achieved in this cohort. This confirms our previous findings that healthcare practitioner interval contributed significantly to diagnostic delay in lymphoma, with a median delay of 48 days from first healthcare contact to diagnostic biopsy and repeat FNAC associated with delay.^1^ A total of 10 patients had repeat sputum tests for Xpert, where an Xpert on FNA would have been more appropriate. Fine needle aspirate for cytology was repeatedly performed and seldom gave a *rule-in* diagnosis, while the poor sensitivity of FNA for either TB or lymphoma made a confident *rule-out* diagnosis impossible. Furthermore, the interval from initial investigation to diagnostic biopsy was also unacceptably long: 49 days (IQR 14–80) for sputum, and 30 days (IQR 6–86) for FNA. Performing a test that is sensitive and specific from the very beginning reduces healthcare utilisation and improves timely diagnosis.^16^

Tests to diagnose tuberculosis adenitis in this patient cohort before biopsy were poorly chosen. Approximately half of the patients underwent FNA cytology, and, in a smaller proportion, a smear for AFBs was requested. Both these tests lack sensitivity for tuberculosis diagnosis because of the paucibacillary setting of lymph node infection.^17,18^ The use of Xpert was confined solely to sputum, and was repeated in one third of cases. This, despite the presence of readily accessible lymphadenopathy. This practice contradicts the 2013 WHO Policy Update on Xpert which clearly recommends Xpert testing of non-respiratory specimens, including lymph node tissue, as the first-line diagnostic test for EPTB.^8,19^ In two diagnostic comparison studies of tuberculosis adenitis, the sensitivity of Xpert (49.3% and 60.1%) was significantly higher than that of smear microscopy (14.5% and 27.8%).^17,20^ More recently, Antel et al. reported 100% specificity and 70% sensitivity for tuberculosis using the Xpert Ultra assay in 43 patients with tuberculosis adenitis.^9^ When patients present with both pulmonary symptoms and significant lymphadenopathy, concurrent Xpert tests (or other TB-NAAT) should be done on both lymph node and sputum. This aligns with recent guidelines that emphasise the ability of concurrent testing to improve diagnostic accuracy.^7^ Lymph node biopsy should be performed timeously, by either core-needle biopsy or surgical excision, if the Xpert is negative, or if the early response to tuberculosis therapy is poor.

Lymphoma accounted for 21% of diagnoses. The use of FNAC in lymphoma diagnosis, which was performed in more than half of the cases, is strongly discouraged because of low test sensitivity, unless auxiliary tests are available to enhance accuracy.^21,22^ A meta-analysis of 42 studies (1989–2012) that reviewed the sensitivity of FNAC to detect lymphoma reported only 12% diagnostic accuracy for lymphoma.^3^ Antel et al. reported an even lower sensitivity, with 11% (*n* = 63/90) of FNACs being merely ‘suggestive’ of lymphoma, and requiring a next step of histological confirmation for final diagnosis.^1^ We confirm this poor performance with FNAC being non-diagnostic in this cohort, and at best one patient with ‘atypical’ FNAC findings. The demographic distribution of lymphoma and EPTB in this cohort justifies the focus on both diagnoses, in the evaluation of lymphadenopathy in patients of any age and any HIV status.^9^

In contrast to the lack of utility of FNAC for lymphoma, the use of FNAC in the solid cancer diagnostic pathway is well established. In patients of an older age, as the importance of metastatic cancer rises, FNAC has an acceptable sensitivity for a first-line investigation.^23^ This sensitivity was not evident in the 15% with solid cancer in our cohort, likely as they were a highly selected group.

**FIGURE 2:**
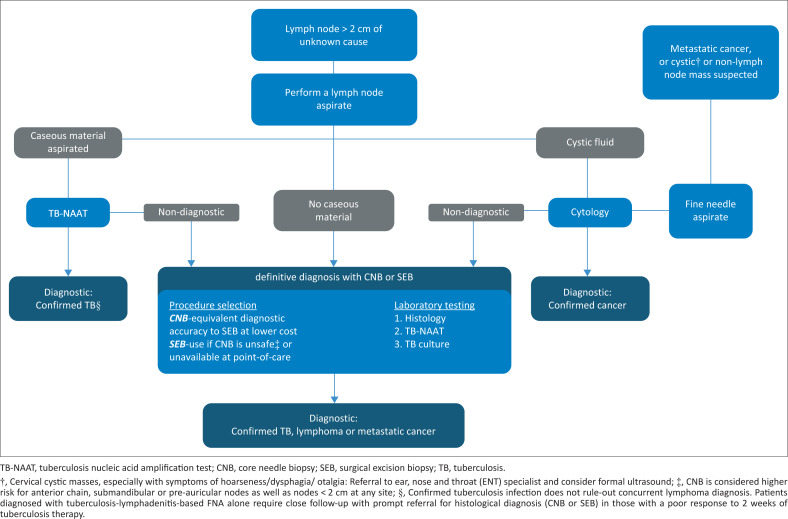
Focused investigative pathways for patients with lymphadenopathy of an unknown cause.

This study highlights the importance of a structured pathway in managing lymphadenopathy to optimise resource use.^21^ Furthermore, this analysis provided the proof of concept for the implementation of the Rapid Access Diagnostic Lymphadenopathy Clinic (RADLAC) at GSH at the end of 2017 – a physician-led clinic centred on the provision of early access to point-of-care core biopsy sent away for both histological analysis and Xpert testing.^1,2,3^ By following the evidence-based diagnostic pathway shown in Figure 2, the RADLAC clinic reduced the healthcare practitioner interval to 27 days.^3^

Our study has limitations. The sample size was small, and was biased through considering a selected cohort of patients in whom diagnosis was not obtained through first-line investigations. Retrospective collection of study data most likely led to an underestimation of the duration of the healthcare practitioner interval. Data were limited to evaluation of visit records, laboratory testing, and documented history given, to ascertain the first date of healthcare intervention, Lastly, all participants had an accessible peripheral lymph node; therefore, our findings may not be generalisable to patients with EPTB or lymphoma with central lymph nodes.

## Conclusion

This retrospective analysis of patients with persistent lymphadenopathy demonstrates how poorly structured diagnostic pathways increase healthcare utilisation and contribute to diagnostic delay in urgently treatable conditions. The median diagnostic interval of 55 days, more than double the UK NICE target of 28 days, results from inappropriate test selection, reliance on FNAC, repeated testing without due consideration of differential diagnoses, and perceived or real barriers to referral for lymph node excision biopsy.

Focused investigations including Xpert and histological examination, are required for at least two key diagnoses in patients with lymphadenopathy, namely tuberculosis and lymphoma. Implementing improved clinical pathways for patients with lymphadenopathy in South Africa’s public healthcare system will enhance timely diagnosis of both tuberculosis and lymphoma, potentially leading to improved patient outcomes.
